# Finnish parents’ perceptions of death following the traumatic death of a child

**DOI:** 10.1186/s40359-024-02220-8

**Published:** 2025-01-14

**Authors:** Nur Atikah Mohamed Hussin, Terhi Talvitie, Eetu Laitinen, Anna Liisa Aho

**Affiliations:** Faculty of Social Sciences (Health), Tampere University, Tampere, Finland

**Keywords:** Perception, Traumatic death, Deaths of child(ren), Grief, Qualitative

## Abstract

Children are expected to outlive and live longer than their parents. However, the traumatic death of a child challenges parents’ understanding of life and death. If parents are unable to form their own perceptions of death after such a loss, it can hinder their ability to cope and adjust. This study aims to explore parents’ perceptions of death following the traumatic death of a child. To recruit participants, an online advertisement was posted on the websites of Finnish bereavement organizations, their member mailing lists, and closed discussion groups. The study consisted of two phases. In the initial phase, 66 parents responded to open-ended questions via the LimeSurvey platform. Subsequently, 17 parents were interviewed in-depth over the phone. The findings were analyzed using thematic analysis. The perceptions of parents who had experienced the traumatic death of a child included death is universal, awakening or preparing for their own death, reunion in death, death and spiritual growth, and death is unjustified. These findings highlight the importance of providing support to parents following the traumatic death of a child, which can help them reconstruct perceptions of death and better adapt to their loss.

## Introduction

The death of a child can lead to profound and enduring grief in parents [1]. Bereaved parents often navigate a complex mix of trauma and grief [2]. The impact of their loss tends to be more severe when the child’s death is due to traumatic causes such as suicide, homicide, violence, or accidents, which can heighten the risk of post-traumatic stress reactions and increase the likelihood of suicide among parents [3]. Before a child is born, parents often envision an idealized future for their child, gradually building hopes and expectations. When their child passes away, parents not only experience emotional and physical loss but also the loss of the envisioned future [4].

The loss of a child defies the natural order, disrupting the expectation that parents will outlive their children [5]. In many cultures, mothers are perceived as protectors and primary caregivers of their children [6–8]. The death of a child is often perceived as a reflection of the mother’s failure, resulting in labels such as “bad mother,” “bad death,” and a curse on the family [6, 7]. In addition, the inability to shield their child from a traumatic death undermines the ideal of parenthood, stigmatizing parents as failures [9]. Consequently, parents struggle to reconcile their belief in destiny with societal values. Various terms, such as “bad death” [7] and “family curse” [6], have been used to describe perceptions of parents after such losses.

Despite perceptions and labeling from society, there is a lack of understanding of parents’ perceptions towards death following the traumatic death of their child. Previous studies have focused on understanding the perceptions of death among children (e.g., [10–12]), culture and religion (e.g. [13]), medical professionals and students (e.g. [14, 15]), and psychology students (e.g. [16]). Perception is a process in which individuals select, organize, and interpret events in their lives [17], and is important for parents to make sense of their loss. Perceptions of death are heavily influenced by culture [18], and also by religion which is also associated with reduced anxiety associated with death [19]. Human culture and associated beliefs, or the rejection thereof, influence how death is perceived [20]. For example, people’s worldviews vary between Eastern and Western cultures, with Eastern cultures generally being seen as more holistic compared to Western cultures.

Several studies have explored parents’ perceptions of death, particularly in cases involving children with chronic illness (e.g. [21, 22]), in general populations (e.g. [23]), and in relation to parental perception of grief intensity within end-of-life and bereavement care (e.g. [24]). Studies report that parents, especially those with children suffering from serious and chronic illnesses, often understand the possibility of their child’s death yet experience intense fear of this loss. Many parents seek ways to cope with their anticipated loss, with some associating death with suffering and linking the location of death to the level of suffering their child may endure.

Additionally, studies on death perceptions have primarily focused on how older people view a good death (e.g. [20, 25]). A systematic review of older people’s perceptions of a good death [20] identified key characteristics such as a dignified moment of death, factors that enhance the will to live, active engagement in adapting to death, and equitable interpersonal relationships. In a study involving South Korean war veterans, perceptions of a dignified death were shaped by maintaining emotional comfort and organizing social relationships. Furthermore, participants’ views on a good death were influenced by their high levels of education and financial stability [25].

Currently, there is a lack of understanding regarding parents’ perceptions of death after the traumatic loss of a child. Thus, it is crucial to conduct studies focusing on these perceptions. Parents’ views on death can significantly influence their ability to cope with such a traumatic event. This study aims to explore parents’ perceptions of death following the traumatic loss of a child. The findings will provide researchers with insights into the unique concepts of death as described by bereaved parents. This understanding can aid professionals in bereavement care settings in supporting and assisting parents with their coping mechanisms and support systems after the traumatic death of a child.

### Finnish perspectives on death

Given that literature indicates perceptions of death are linked to culture and religious beliefs, it is valuable to discuss Finnish backgrounds and cultural perspectives related to death [19, 18]. In Finland, the secular notion of death is widely accepted, but some also embrace the idea of reunion in death [26]. This concept can be broken down into two distinct categories: supernatural and naturalistic. Believers in a higher power tend to focus on the supernatural, often referencing religious terminology and the immortality of the soul. However, those who do not believe in a deity or are uncertain in their belief tend to favor a more naturalistic view of continuity [26] and often use science-related terminology to describe the process of bodily decay and the cycle of nature. Even though some Finns may not believe in religion and lean toward a more naturalistic view, it is not accurate to label them as atheistic. Despite a decline in religious practices among Finns [27] they still hold a positive attitude toward their religion. It is more appropriate to describe Finns as ‘belonging without practicing,’ reflecting a secular notion [27]. In a way, though Finns reported not believing in life after life and evil [27], Finns also have a positive attitude toward churches and are described as considering the turn to culture, history, and heritage as a strategy for the preservation of majoritarian ‘religious’ practices [27].

Despite the ongoing research on death perceptions, there is still a dearth of knowledge when it comes to specific populations such as parents who have experienced the traumatic death of a child. The importance of this study lies in the fact that parents may have unique perspectives on death given the prevalence of such traumatic events in their lives.

### Aims

This study aims to explore parents’ perceptions of death following the traumatic loss of a child, addressing a gap in the literature regarding this critical and complex experience. Our research focuses specifically on parents’ perspectives in the aftermath of such a traumatic event, seeking to deepen the understanding of their experiences and the ways they interpret death after the loss of a child.

## Methods

### Research design

This qualitative descriptive study is a component of a broader mixed methods research project examining the grief and bereavement experiences of parents whose children died from traumatic incidents. The study employs phenomenological research design. As described by [28], phenomenological studies explore the everyday lived experiences of individuals, emphasizing their shared experiences when encountering a specific phenomenon. According to [29], phenomenology enables researchers to interpret the meaning and nature of experiences within a particular group by sharing their narratives. The primary goal of phenomenology is to extract the essence of the experiences under investigation, capturing the core nature of the phenomenon [28]. Researchers aim to understand this essence concerning human nature and common lived experiences [28].

### Data collection and participants

The study was advertised on the member mailing lists, closed discussion forums, and websites of grief organizations in Finland. The advertising included information on the objectives and purpose of the study, together with contact information and a link to an electronic questionnaire created with LimeSurvey. Each of those who responded also received a survey and a cover letter relating to the study. The study invited participants who fulfilled the inclusion criteria, including parents who had experienced the traumatic loss of a biological child. There was no restriction placed on the deceased child’s age for this study.

The electronic questionnaire was created using the LimeSurvey platform and was available in Finnish and English. A variety of demographic background data, including age, sex, cause of death, time since loss, number of surviving children, perceived health state, and informed consent information were included in the questionnaire. The central questions asked were “How do you perceive death following the traumatic death of your child? Explain how the loss could have affected your perspective on death and dying”.

Participants had the opportunity to indicate their interest in taking part in a phone interview after completing the electronic questionnaire. The contact details of those who expressed interest and consented to take part in the interview were taken. The participants were then contacted by researchers via text or email to schedule a convenient time for the interview.

The open-ended questions from the electronic questionnaire were also used in the telephone interviews, which were conducted in Finnish. These interviews served as a means for participants to expound on the answers they had provided in the electronic questionnaire they had completed before the interview. The interviews were recorded once the participants had granted their permission. After the interview was completed, the interviewer switched off the recording device and had an informal discussion with the parents. The purpose of the informal discussion was to ensure that the parents were not experiencing any psychological distress following their interviews, and if they were, they could take the opportunity to let the researchers know. Nevertheless, none of the parents claimed to be experiencing psychological distress following the interview. The interviews lasted for a duration of anything between 45–90 min.

In total, 66 participants answered the electronic questionnaire. The participants were between 30–71 years old (av. 48 years). The majority of parents (80%) were married or in a relationship, and the majority (89%) had living children. Slightly under one-half of the parents belonged to a religious community (47%). At the time of answering the survey, one to five years (21.2%) more than five years (39%), or more than five years (39%) had passed since the death of the child. The most common causes of the child’s death were suicide (27%), traffic accidents (24%), and accident or sudden death (20%). The demographic profiles of the questionnaire participants are presented in Table 1.

**Table 1:** List of demographic characteristics of the participants from the LimeSurvey questionnaire

Background variable	n (66)	%
**Sex (*n* = 66)**		
Female	65	98.5
Male	1	1.5
**Age in years (*n* = 66)**		
Less than 40	9	13.6
40–49	14	21.2
50–59	30	45.5
≥ 60	11	16.7
Do not want to disclose	2	3.0
**Marital status (*n* = 66)**		
Married	42	63.6
In a relationship	11	16.7
Divorced or widowed	13	19.7
**Residency areas (*n* = 66)**		
Southern Finland	25	37.9
Western Finland	21	31.8
Eastern Finland	10	15.2
Northern Finland	10	15.2
**Educational background (*n* = 65)**		
Comprehensive school	3	4.5
Upper secondary vocational qualification	23	34.8
University of Applied Sciences degree	20	30.3
Academic degree	19	28.8
Do not want to disclose	1	1.5
Employment status (*n* = 66)		
Employed	43	65.2
Unemployed, on sick leave or retired	15	22.7
Students or others	8	12.1
**Member of religious community (*n* = 66)**		
Yes	31	47.0
No	35	53.0
**Perceived well-being (*n* = 66)**		
Poor	14	22.2
Satisfactory	24	38.1
Good	25	39.7
Do not want to disclose	3	4.5
**Number of surviving children (*n* = 66)**		
0	7	10.6
1–2	38	57.6
≥ 3	21	31.8
**Cause of death of child (*n* = 66)**		
Suicide	18	27.2
Traffic accident	16	24.2
Other types of accidental deaths such as falling from horse riding, choking in bed, etc	13	19.7
Sudden death	13	19.7
Violence / other external cause	6	9.1
**Time elapsed since the death of the child in years (*n* = 66)**		
Less than 1	14	21.2
1–5	26	39.4
More than 5	26	39.4

A total of 17 participants participated in the telephone interviews. Most of the participants were women (87%). The most common cause of death for a child was suicide (35%). The majority of parents had five or more years (69%) since the death of their child when participating in the interview. The age at death of the child varied from under one year old to almost 30 years old. The demographic profiles of the interview participants are presented in Table 2.

**Table 2:** List of demographic characteristics of the participants from the interview

Theme	Categories	Number (*N* = 17)
Sex	Female	15
	Male	2
Cause of death of a child	Suicide	7
	Accident	5
	Homicide	1
	Medical malpractice	2
	Sudden	2

### Trustworthiness

Four key factors—credibility, confirmability, dependability, and transferability—are used to assess the trustworthiness of this study [30]. Credibility was achieved through member checks, involving both researchers in the process. To ensure the study’s transferability to similar populations, a purposeful sampling strategy was employed, selecting participants who could provide rich information about their experiences of losing a child due to traumatic death. Confirmability was accomplished through techniques such as bracketing interviews and reflexivity. Confirmability of qualitative data is ensured by continuously checking and rechecking data throughout the collection process. It can be documented with a clear coding schema that identifies the codes and patterns found in analyses, a technique known as an audit trail. Additionally, confirmability is supported through triangulation, member checking, and reflexive practices to address potential personal biases.

### Ethics approval and consent to participate

This study focused on sensitive social research topics, particularly those surrounding grieving parents. Thus, to ensure the protection of the welfare, rights, and dignity of the study participants, ethical approval was necessary. This study (76/2022) has been approved by the ethical committee of Tampere University.

All of the study participants were asked to give verbal and online consent before beginning the study and were supplied with detailed information about the study objectives. It is important to note that before participating in the study, each subject gave their consent, and participants only responded to the electronic questionnaire and took part in the interviews having completed the informed consent procedure. Consequently, informed consent was obtained from all participants in the study.

Regarding the principles for ethical research conduct, the guidelines developed by the Finnish National Board on Research Integrity [31] were adhered to throughout the study. Confidentiality was maintained regarding the parents’ data, which was only utilized for the identified purpose. On the secure LimeSurvey platform, demographic data is kept confidential, and upon completion of the study, all the gathered data will be securely deleted.

Debriefing with the participants was conducted by the researcher to ensure the participants’ well-being was preserved, and to provide them an opportunity to reflect on their interview experience. Parents were offered helpful advice and urged to ask for help and support when they required it.

### Data analysis

The open-text responses from the electronic LimeSurvey survey were initially transferred to an Excel database for analysis. Exporting responses into a compatible database helps eliminate transcription errors and prevents participants from modifying their responses [32]. Data from audiotaped interviews were then transcribed verbatim. Both data sources were combined into a single document and translated from Finnish to English by a professional translator, as one of the researchers does not speak Finnish. This translation allowed both researchers to collaborate effectively during data analysis.

Both researchers, who each have over 10 years of experience in grief and loss research, participated in the data analysis. One researcher has a nursing background, and the other has a social work background, providing diverse perspectives on the topic. The computer-based analysis tool ATLAS.ti was used for data analysis. Thematic analysis was employed to identify recurring patterns across participants’ subjective responses [33]. The data was analyzed using a six-step thematic process: familiarization, coding, theme generation from codes, theme review, theme definition and naming, and write-up [33]. This process was guided by their quality checklist to ensure rigor and reliability.

The analysis involved multiple readings of the transcripts to familiarize the researchers with the data. Meaningful units were identified, categorized, and labeled as codes, which were then grouped based on similar content. Codes and groups were continually compared and cross-referenced among the interview transcripts. A triangulation approach ensured the credibility of the analysis [34]. The researchers regularly discussed their interpretations, and agreement from both was necessary to confirm the codes, categories, and groups derived from the interviews. If disagreements occurred, they discussed the data until a consensus was reached, ensuring all themes were supported by quotes from participants. Data collection continued until saturation was achieved and no new themes emerged.

### Positionality of the researcher and reflexivity in the study

Moos [35] observed that definitions and understandings of grief vary based on the researcher’s perspective. In this study, one researcher has personal experience with miscarriage, while others have no experience with parental grief. However, our experience working with bereaved individuals and conducting grief-related research provided a professional perspective that informed our discussion.

Parental perceptions of death are highly individual. Although the literature notes that death perceptions often align with cultural backgrounds, we observed this as well. Some parents in this study viewed death through a religious lens, a perspective discussed in Finnish grief literature. While each parent’s view of death is shaped by personal experience, this study highlights the importance of further research on post-traumatic growth following the loss of a child, as it may be closely linked to parents’ perceptions of their child’s death.

## Results

Five specific themes emerged from the data: death is universal, awakening or preparing for their own death, reunion in death, death and spiritual growth, and death is unjustified.

### Death is universal

For some parents, death was seen as inevitable. Death is described as an event that can happen to anyone at any time. A 33-year-old mother who lost her child due to an accident commented:

*My attitude towards death hasn’t changed. I have always known that anyone can die at any time and that human destinies are not always fair.*

Death is universally recognized as an inevitable part of life. While parents often perceive death as something that only happens to older people, the sudden loss of a loved one can profoundly challenge their sense of security. A 38-year-old mother who lost her child due to an accident commented:

*Death has become an “ordinary” thing. In the past, I have relied on death in old age or illness. Sudden death, in a second, erodes the foundation of life and takes away the sense of security.*

Some parents recognize that death is a universal event affecting everyone. They explain that, in reality, every individual is ultimately waiting for their time to die. A mother who lost her child due to an accident shared:

*We all die and get to the other side. I live to the end of my earthly life, and then I move on to the other side. I work with the elderly. I understand when they are waiting for their own death. It’s natural.*

For some parents, the thought of death can be anxiety-inducing, as it represents the final stage of life. When they were younger, they didn’t think much about death. However, experiencing a loss makes them realize that death is universal and that nothing lasts forever. A 66-year-old mother who lost her child due to a homicide said:

*I have to admit that death is haunting when it’s so final, after that it’s all… The hardest part is, although I believe so, fear and anxiety are present from time to time. When I was younger, I didn’t think about death that way, but through my own losses, I have become limited and become aware that nothing is eternal.*

Additionally, experiencing past losses helps parents understand that death is a universal part of life. A 68-year-old mother who lost her child due to suicide commented:

*Since I was a child, I have buried my brother, mother, and father, so coffins and funerals have become familiar. I have realized that at birth we also make death a part of our lives.*

Therefore, some parents concluded that death and life are correlated. A mother who lost her child due to malpractice shared:

*We are born to die, that is, we are born with a birth gift of death at our side. Together with a friend who also lost her son to suicide, we talked about how natural death is.*

### Awakening or preparing for their own death

Parents reported that their fear of death diminishes over time, although they expressed concern about losing their other children. They sought to find peace with the concept of death by accepting that they, too, would eventually pass away. A 49-year-old mother who lost her child to an accident shared:

*With death I am you today, I am not afraid of it. And sometimes, when it’s my time, I will get to be next to my child. The fear is just that one of my other children will die. It has changed my perception of death completely.*

Additionally, some parents experience a profound awakening, leading them to prepare for their own passing, including making funeral arrangements. This also allowed them to share their plans with their loved ones. A 63-year-old mother who lost her child to suicide commented:

*I actively think of and fear death all the time. I visit my child’s grave every day. I have purchased my own urn and coffin and made a preliminary agreement with the funeral home. My surviving child knows about this.*

Eventually, for some parents, the cemetery is no longer a fearful place to go but rather a peaceful and safe place. Parents cherished every moment they had with their deceased children. A 55-year-old mother who lost her child due to malpractice said:

*I used to be afraid of going to the cemetery. When my child was buried, the cemetery became a more peaceful and somehow safe place. The concept of death; My children and I have gone through many different phases.*

### Reunion in death

For some parents, the death of their child does not signify the end of their relationship but rather represents a hopeful anticipation of reunion in the afterlife. A 53-year-old mother who lost her child to suicide said:

*I have family here, and a daughter out there somewhere. When I die, I will continue living with her.*

Similarly, parents may perceive death as an opportunity for reunion with their deceased child, providing them with a sense of comfort. A 51-year-old mother who lost her child to an accident said:

*The death of a child drastically changed the idea [of death]. I believe that you have to live as well as you can before your own. Sometimes, amid great grief, I wished I would die so that I could be reunited with my child. I chose to live and live. Death is sometimes perhaps a comforting friend to receive.*

Similarly, parents reflected on their relationships with both their deceased and surviving children. The bond with the deceased child continues beyond the parents’ death, while, during their lifetime, they cherish the moments spent with their surviving children. A 48-year-old mother who lost her child to an accident maintained:

*After the death of my child, I am much calmer about my own mortality. When it’s time for me to go, then I’ll go to my dead child. And as long as I get to live, I will spend that time with my surviving children.*

### Death and spiritual growth

For some parents, the experience of death is perceived as an opportunity for spiritual growth, allowing them to re-evaluate their lives. Following the traumatic death of a child, parents re-evaluate their overall life values. Parents shared their thoughts that it is unnecessary to try to find explanations for every event in life, but more importantly to focus on reconstructing life values. A father who lost his child to suicide said:

*I had thought about it before, but in this situation, of course, I had to rethink my life values overall. This is our smallness in this universe, though it is kind of out of control. Not everything can necessarily be justified by reason, and there is certainly no rational explanation for everything.*

Death encourages parents to gain a deeper appreciation for themselves and their lives. Life is described as a valuable gift that parents should appreciate. A 56-year-old mother who lost her child to homicide shared:

*After the sudden death of the child, I hoped for years that I would get out as quickly as possible. It wasn’t until I realized that I didn’t appreciate God’s gift to me, my own life. The gift from God - my child, took precedence over everything. And it hurt so much when he had to give up, that he couldn’t appreciate his gift of life.*

Likewise, for some parents, death is perceived as an opportunity for parents to be more present and appreciative towards their surviving children and their lives. Noting that illness can happen at any time, life should not be taken for granted. A mother who lost her child to malpractice shared:

*After the death of a child, death has become more real and the value of the surviving children and life increases. Nothing is taken for granted anymore, and I strive to live in the moment if possible. The trauma caused me to be afraid of illness and I tried to make my dreams come true as soon as possible.*

### Death is unjustified

After the traumatic death of a child, some parents viewed death through a fatalistic lens. Struggling to make sense of their loss, they associated death with bad luck. One mother, whose child died due to malpractice, expressed her feelings by saying:

*Maybe I see the death of a child as very bad luck. This just happens to someone, or our family can’t rationally think that there is a special reason or explanation for it. A bad coincidence and the sum of events.*

Parents shared that their spiritual journey in making sense of the traumatic death of a child is fraught with challenges. This experience often leads them to perceive the loss as unjust and to question why God would allow them to endure such profound pain. A 56-year-old mother who lost her child due to an accident shared:

*Death is always final, unfortunately, I can’t believe in any reunion, I used to pursue religion in my own life as well, but after my daughter’s death, the question has arisen as to what kind of God it is who wants to inflict such pain.*

Additionally, while the death of older individuals is often seen as a natural part of life, many parents perceive the death of young people as tragic and unjustified. A 48-year-old mother who lost her child to suicide shared her thoughts of having difficulties in making sense of the death of young people, compared to those of older people. She said:

*The deaths of elderly people feel more natural, and I cannot see anything extraordinarily sad about them. The death of a young person, on the other hand, is such an extraordinarily tragic matter.*

## Discussion

This qualitative study aims to explore parents’ perceptions of death following the traumatic death of a child. The findings suggest that parents’ perceptions of death vary in this context. According to the literature, individuals can perceive death as a universal phenomenon that affects all living things [36]. The findings of this study align with previous research, indicating that parents also perceive death as a universal concept.

Parents’ preliminary understanding of death, including previous losses and working experience, shape their perceptions of death. Unlike perceiving death as something that primarily affects older people or individuals with sickness, the death of a child at a young age makes parents realize that death can happen to anyone, without any specific reason. Despite the immense pain experienced by the parents in this study due to the traumatic death of their child, they still perceive death as a natural and essential part of life. Parents eventually reconcile with the loss and reconstitute their lives [37].

According to [38], around midlife individuals typically begin to turn inward to explore the more spiritual aspect of the self. However, this study does not attempt to confirm this finding or compare the spirituality of individuals of different ages. Parents in this study described death as an awakening experience, one that blends cognitive, reflective, and emotional aspects of their personalities [39]. They perceive death as an awakening experience by embracing various perspectives that help them transcend subjectivity and personal projections (reflective dimension) while uncovering the deeper meanings behind phenomena and events (cognitive dimension). Contrary to the literature that suggests death-related experiences can increase an individual’s anxiety about death [19, 40], this study finds that parents’ fear of death diminishes. Parents have developed an awakened perspective toward their own mortality, recognizing it as an ongoing process of adaptation [41]. Parents in this study are more open [42] to discussing death, including their own preparations such as funeral planning, with loved ones. Although the literature on this topic is limited, discussing and planning for one’s own death can improve coping mechanisms [43]. This study suggests that facilitating discussions about death with parents after the traumatic loss of a child may help them develop a personal perception of death, thereby enhancing their coping and adaptation in the aftermath.

In addition, instead of fearing death, parents focused on the possibility of being reunited with their deceased child in the afterlife. A study on afterlife beliefs found that these beliefs are shared by both religious and non-religious individuals [44]. Additionally, belief in the afterlife is a well-known aspect of Nordic culture [42]. Although Finnish parents described themselves as non-religious, this study’s findings have discovered a theme of reunion with the deceased in afterlife. This suggests that the idea of reunion may or may not be related to religiosity, but rather to individual perceptions of death. It could also be understood that the idea of reunion in parents reflects the cultural reflections of Finns, which may have been influenced by previous religious teachings on the concept of an afterlife. Instead of viewing the loss as a separate experience, parents perceived the death of their child as a continuation of their own lives. According to [45] “internalizing” the lost loved one as an extension of oneself helps enhance emotional regulation by maintaining a psychological closeness to the attachment figure, rather than a physical one. This involves viewing the deceased as a role model, valuing their unique legacy, or feeling their comforting presence during times of stress. Therefore, parents internalize the loss of their child as an extension of themselves, which can later provide comfort as they believe in being reunited with their deceased child in the afterlife. In this study, parents expressed their desire to be reunited with their deceased child through spiritual means and felt a connection with them. This perception of death as a way to reconnect spiritually helps parents continue their lives and be more prepared for their own eventual death.

The study found that parents understood that they cannot control every aspect of life, including death. This realization prompted them to reevaluate their perspective on life and view death through their own lens. Furthermore, parents expressed a sense of calmness regarding death and an increased appreciation for their own lives and the lives of others, particularly their surviving children. It is worth noting that gratitude toward life is often associated with appreciation [46]. In this study, parents reported a heightened sense of gratitude toward life following their loss. Interestingly, the topics of grief and gratitude toward life are not extensively discussed in the existing literature [47]. However, a cross-cultural study conducted by [48] revealed that spiritual practices among Asian and African participants promoted a sense of gratitude toward life, which in turn influenced their perceptions of loss.

Nonetheless, parents often face a significant challenge to their fundamental belief that the world is a safe place when they experience the traumatic death of a child [49]. According to a study, parents may find it difficult to comprehend their losses, leading them to perceive death as unjustified. Interestingly, some parents even view death as bad luck. Although rare, a study conducted in Finland on traffic deaths and superstition found that some Finns believe in superstitions, such as the idea that Friday the 13th can be dangerous for certain women and potentially lead to their death [50]. The belief in luck reflects a perception that individual events are externally triggered, uncontrollable, irrational, and have little influence on future expectations [51]. However, individuals who attribute events in their lives to luck are also more likely to experience a decline in mental health [51]. Consequently, the parents in this study also reported feeling that death is unjustified and challenging to comprehend, which can harm their mental well-being. Parents who perceive death as unjustified may face additional difficulties in accepting their loss. It is important to note that this study did not quantify these relationships, but it does highlight the need for future research to thoroughly investigate negative perceptions of death, including how bereaved individuals cope and the outcomes of their grief [49].

The traumatic death of a child heightened parents’ anxiety about the future; however, they also recognized that death is an inevitable part of life that cannot be prevented. Parents understand the importance of moving forward in their lives despite their grief. Following the traumatic loss of a child, parents’ perceptions of death are significantly altered. Although this study did not aim to compare changes in perceptions of death between traumatic and natural causes, it suggests that understanding parents’ perceptions is crucial and can lead to deeper insights into the complex and ongoing nature of grief.

## Conclusion

The findings of this study underscore the complexities of understanding parents’ perceptions of death, which are influenced by various factors, including their prior experiences with loss. Notably, the traumatic death of a child can profoundly shape these perceptions. While death is a universal aspect of human existence, the impact of traumatic loss can be particularly harsh for parents. Although parents’ perceptions of death may or may not be influenced by the cause of death, it is evident that those who experience the traumatic loss of a child may view their loss as a matter of bad luck, which can subsequently affect their overall well-being.

Acknowledging and discussing death with parents is crucial for supporting them through the grieving process. This study highlights the importance of providing support to help parents in their grief, enabling them to develop their own understanding and perception of death. To deepen understanding of this topic, further studies are needed to explore the perception of death among different grieving populations.

### Limitations of study and future studies

To recruit participants for the study, advertisements were posted on Finnish peer-support websites. This method may have excluded parents who could not access these websites. However, using an internet platform allowed participation from a more geographically diverse group of interested parents, even though the study was nationally focused. Future research should explore alternative data collection methods to encourage participation from parents who are not involved with peer-support organizations and have limited support systems.

The study used the online LimeSurvey questionnaire for data gathering, which could have been problematic for parents without internet access. To mitigate this, the study team communicated with interested parents over the phone, reducing exclusion and fostering a more inclusive participation process. Phone communication can also alleviate concerns of being recognized by parents, which might otherwise deter participation. Future studies should consider face-to-face interviews to compare different data collection methods and their impacts. Furthermore, this study employed thematic analysis, which limits the ability to quantify content and themes. However, this does not diminish the quality of the findings. Additional research is needed to provide quantitative insights into this topic.

It is also noted that the study primarily involved female participants, highlighting the need for further research focusing on fathers who have experienced the traumatic loss of their children. Additional studies should aim to ensure better participation from male participants for a more balanced understanding of this topic. Lastly, this study did not collect data on the age of the deceased child, as it did not aim to compare the effects of grief based on the child’s age at death. Future research should include this factor to examine whether the age of the deceased child influences the parents’ grief.

## Data Availability

No datasets were generated or analysed during the current study.

## References

[1] Hussin NAM, & Aho AL. Meaning-making Coping in Finnish Parents Following the Traumatic Death of a Child. Illn Crisis Loss. 2024. 10.1177/10541373241256452.

[2] Raphael B. Grieving the death of a child. BMJ. 2006;332(7542):620–1. 10.1136/bmj.332.7542.620. 16543305 10.1136/bmj.332.7542.620 PMC1403238

[3] Murphy SA. A bereavement intervention for parents following the sudden, violent deaths of their 12–28-year-old children: Descriptions and applications to clinical practice. Can J Nurs Pract. 1997;29(4):51–72 PMID: 9697435. 9697435

[4] Michon B, Balkou S, Hivon R, Cyr C. Death of a child: Parental perception of grief intensity - End-of-life and bereavement care. Paediatr Child Health. 2003;8(6):363–6. 10.1093/pch/8.6.363. 20052330 10.1093/pch/8.6.363 PMC2795457

[5] Najafi K, Shirinabadi Farahani A, Rassouli M. et al. Emotional upheaval, the essence of anticipatory grief in mothers of children with life-threatening illnesses: a qualitative study. BMC Psychol. 2022;10:196. 10.1186/s40359-022-00904-7. 10.1186/s40359-022-00904-7 PMC9366755 35953867

[6] Fu F, Chen L, Sha W, Chan CLW, Chow AYM, Lou VWQ. Mothers’ Grief Experiences of Losing Their Only Child in the 2008 Sichuan Earthquake: A Qualitative Longitudinal Study. OMEGA J Death Dying. 2020;81(1):3–17. 10.1177/0030222818755287. 10.1177/0030222818755287 29380658

[7] Ismail A. Arab Muslim Mothers’ Struggle Attributing Meaning to Child Loss in Home Accidents. J Loss Trauma. 2022;27(7):642–60. 10.1080/15325024.2021.2013673.

[8] Kim Y, Lee D, Jeon H. A Longitudinal Perspective on Bereaved Parents’ Changes in Life Experience after the 2014 Sewol Ferry Sinking. Omega J Death Dying. 2022;85(3):520–53. 10.1177/0030222820947238. 10.1177/0030222820947238 32772640

[9] Huggins C, Hinkson G, Charles K. “He Was a Good Boy”: The Caribbean Black Mothers’ Experience of Coping and Grief with the Homicide of Their Sons in Trinidad. J Black Stud. 2020;51(5):411–32. 10.1177/0021934720915441.

[10] Ahmadi F, Ristiniemi J, Linblad I, Schiller L. Perceptions of death among children in Sweden. Int J Child Spiritual. 2019;24(4):415–33. 10.1080/1364436X.2019.1672627.

[11] Ellis JB, Stump JE. Parents’ perceptions of their children’s death concept. Death Stud. 2000;24(1):65–70. 10.1080/074811800200702. 10915448 10.1080/074811800200702

[12] Gutiérrez IT, Menendez D, Jiang MJ, Hernandez IG, Miller P, Rosengren KS. Embracing Death: Mexican Parent and Child Perspectives on Death. Child Dev. 2020;91(2):e491–511. 10.1111/cdev.13263. 31140591 10.1111/cdev.13263 PMC6883122

[13] Okechi OS. Culture, Perception/Belief about Death and their Implication to the Awareness and Control of the Socio-Economic, Environmental and Health Factors Surrounding Lower Life Expectancy in Nigeria. Acta Psychopathol. 2017;3:56. 10.4172/2469-.

[14] Asadpour M, Sabzevari L, Ekramifar A, Bidaki R. The Attitude of Medical Students Toward Death: A Cross-Sectional Study in Rafsanjan. Indian J Palliat Care. 2016;22(3):354–61. 10.4103/0973-1075.185084. 27559268 10.4103/0973-1075.185084 PMC4973500

[15] Hamadeh RR, Abuelaish I, Yousufzai SJ, et al. Knowledge and attitudes of medical students toward death: a cross-sectional comparative study between an Arab and a Western University. BMC Psychol. 2024;12:133. 10.1186/s40359-024-01616-w. 38459586 10.1186/s40359-024-01616-w PMC10924328

[16] Testoni I, Iacona E, Corso C, Pompele S, Dal Corso L, Orkibi H, Wieser MA. Psychology Students’ Perceptions of COVID-19 in a Death Education Course. Front Public Health. 2021;9:625756. 10.3389/fpubh.2021.625756. 33937167 10.3389/fpubh.2021.625756 PMC8086793

[17] Fiske ST, Taylor SE. Social cognition. Reading, MA: Addison-Wesley; and Schiffman, H. R. (1990). Sensation and perception: An integrated approach. New York, NY: Wiley; 1991.

[18] Tungjitcharoen W, Berntsen D. Afterlife future thinking: imagining oneself beyond death. Mem Cognit. 2023;51(1):4–22. 10.3758/s13421-022-01308-z. 35415796 10.3758/s13421-022-01308-z

[19] Mak M. Quality insights of university teachers on dying, death, and death education. Omega J Death Dying. 2012;66(2):173–94. 10.2190/om.66.2.e. 10.2190/om.66.2.e 23472324

[20] Järviö T, Nosraty L, Aho AL. Older individuals’ perceptions of a good death: A systematic literature review. Death Stud. 2023;47(4):476–89. 10.1080/07481187.2022.2092787. 35775466 10.1080/07481187.2022.2092787

[21] Farthing P, Bally J, Rennie DC. Perceptions Related to Death in Adolescents and Their Parents During the Management of Type 1 Diabetes: A Thematic Analysis. J Pediatr Health Care. 2024;2024(38):586–94. 10.1016/j.pedhc.2024.04.002. 10.1016/j.pedhc.2024.04.002 38661590

[22] Broden EG, Mazzola E, DeCourcey DD, Blume ED, Wolfe J, Snaman JM. The roles of preparation, location, and palliative care involvement in parent-perceived child suffering at the end of life. J Pediatr Nurs. 2023;72:e166–73. 10.1016/j.pedn.2023.06.024. 37355461 10.1016/j.pedn.2023.06.024

[23] Ellis JB, Stump JE. Parents’ perceptions of their children’s death concept. Death Stud. 2000;24:65–70. 10.1080/074811800200702. 10915448 10.1080/074811800200702

[24] Michon B, Balkou S, Hivon R, Cyr C. Death of a child: Parental perception of grief intensity - End-of-life and bereavement care. Paediatric Child Health. 2003;8(6):363–6. 10.1093/pch/8.6.363. 10.1093/pch/8.6.363 PMC2795457 20052330

[25] Hong M, Hong S, Adamek ME, Kim MH. Death Attitudes Among Middle-Aged Koreans: Role of End-of-Life Care Planning and Death Experiences. Int J Aging Hum Dev. 2018;86(1):51–68. 10.1177/0091415016689473. 28105867 10.1177/0091415016689473 PMC5511773

[26] Haimila R, Muraja E. A Sense of Continuity in Mortality? Exploring Science-Oriented Finns’ Views on After Death. Omega J Death Dying. 2023;88(1):38–65. 10.1177/00302228211038820. 10.1177/00302228211038820 PMC10568951 34407669

[27] Taira T. More visible but limited in its popularity Atheism (and atheists) in Finland. New Visibility Atheism Eur. 2012;2(1):21-35. 10.30664/ar.67489.

[28] Creswell JW, Poth CN. Qualitative inquiry and research design: Choosing among five approaches (4th ed.). Thousand Oaks: Sage Publishing, Inc; 2018.

[29] Deepa R, Panicker A. S A Phenomenological Approach to Understand the Challenges Faced by Medical Students. Qual Rep. 2016;21(3):584–602. 10.46743/2160-3715/2016.2222.

[30] Lincoln YS, Guba EG. Naturalistic inquiry. Sage. 1985. 10.1016/0147-1767(85)90062-8.

[31] TENK, Finnish National Board on Research Integrity. Responsible conduct of research (RCR). 2023. Retrieved from: https://tenk.fi/fi/tiedevilppi/hyva-tieteellinen-kaytanto-htk.

[32] Ilieva J, Baron S, Healey NM. Online Surveys in Marketing Research. Int J Mark Res. 2002;44(3):1–14. 10.1177/147078530204400303.

[33] Braun V, Clarke V. Using thematic analysis in psychology. Qual Res Psychol. 2006;3(2):77–101. 10.1191/1478088706qp063oa.

[34] Nowell LS, Norris JM, White DE, Moules NJ. Thematic analysis: striving to meet the trustworthiness criteria. Int J Qual Method. 2017;2017(16):1609406917733847. 10.1177/1609406917733847.

[35] Moos NL. An integrative model of grief. Death Stud. 1995;19(4):337–64. 10.1080/07481189508252737.

[36] Speece MW, Brent SB. The “Adult” Concept of Death: Irreversibility. s.l. Philadelphia: The Charles Press Publishers; 1991.

[37] Christ G. Healing children’s grief: Surviving a parent’s death from cancer. Oxford: Oxford University Press; 2000.

[38] Jung CG. On the psychology of the unconscious. In H Read, M Fordham, & G Adler (Eds.), Jung: Collected Works (Vol. 7). Princeton University Press. 1943.

[39] Ardelt M. Wisdom and life satisfaction in old age. J Gerontol Psychol Sci. 1997;52B:P15–27. 10.1093/geronb/52B.1.P15. 10.1093/geronb/52b.1.p15 9008672

[40] Kastbom L, Milberg A, Karlsson M. A good death from the perspective of palliative cancer patients. Support Care Cancer. 2017;25(3):933–9. 10.1007/s00520-016-3483-9. 27837324 10.1007/s00520-016-3483-9

[41] Elfers J, Hlava P, Sharpe F, et al. Resilience and Loss: The Correlation of Grief and Gratitude. Int J Appl Posit Psychol. 2024;9:327–45. 10.1007/s41042-023-00126-1.

[42] Haraldsson E. Popular psychology, belief in life after death and reincarnation in the Nordic countries. West East Eur Nordic Psychol. 2006;58(2):171–80. 10.1027/1901-2276.58.2.171.

[43] Cruse Bereavement Support. Planning for death: National attitudes towards death, dying, bereavement and later life planning. 2023. Retrieved from https://downloads.ctfassets.net/iqbixcpmwym2/4tUWXfUKLoDOkspbj9ibYy/624a094c576b564204ecbc630d6c29ba/Co-op_P.

[44] Reece GA, Van Tongeren DR, Van Cappellen P. Eternal outgroups: Afterlife beliefs predict prejudice. Personal Individ Differ. 2023;214:1–12. 10.1016/j.paid.2023.112352.

[45] Field NP, Gao B, Paderna L. Continuing bonds in bereavement: An attachement theory based perspective. Death Stud. 2005;29:277–99. 15849880 10.1080/07481180590923689

[46] Adler MG, Fagley NS. Appreciation: Individual differences in finding value and meaning as a unique predictor of subjective well-being. J Pers. 2005;73(1):79–114. 10.1111/j.1467-6494.2004.00305.x. 15660674 10.1111/j.1467-6494.2004.00305.x

[47] Ramstad S. Grief and Gratitude. Creat Nurs. 2014;20(3):194–6. 10.1891/1078-4535.20.3.194. 25252384 10.1891/1078-4535.20.3.194

[48] Popovich PA. Cultivating gratitude after loss: A cross-cultural, mixed-methods research approach. Palo Alto: Institute of Transpersonal Psychology; 2014.

[49] Park CL. Making sense of the meaning literature: An integrative review of meaning-making and its effects on adjustment to stressful life events. Psychol Bull. 2010;13:257–301. 10.1037/a0018301. 10.1037/a0018301 20192563

[50] Näyhä S. Traffic deaths and superstition on Friday the 13th. Am J Psychiatry. 2002;159(12):2110–1. 10.1176/appi.ajp.159.12.2110. 12450968 10.1176/appi.ajp.159.12.2110

[51] Rotter J. Generalized expectancies for internal versus external control of reinforcements. Psychol Monogr. 1966;80:609. 10.1037/h0092976. 5340840

